# SARS-CoV-2, ACE2, and Hydroxychloroquine: Cardiovascular Complications, Therapeutics, and Clinical Readouts in the Current Settings

**DOI:** 10.3390/pathogens9070546

**Published:** 2020-07-07

**Authors:** Rajkumar Singh Kalra, Dhanendra Tomar, Avtar Singh Meena, Ramesh Kandimalla

**Affiliations:** 1 AIST-INDIA DAILAB, DBT-AIST International Center for Translational & Environmental Research (DAICENTER), National Institute of Advanced Industrial Science & Technology (AIST), Higashi 1-1-1, Tsukuba 305 8565, Japan; 2 Center for Translational Medicine, Lewis Katz School of Medicine, Temple University, Philadelphia, PA 19140, USA; 3 CSIR-Centre for Cellular and Molecular Biology (CCMB), Habsiguda, Uppal Road, Hyderabad 500 007, Telangana State, India; avtar.ccmb@yahoo.com; 4 Applied Biology, CSIR-Indian Institute of Chemical Technology (IICT), Uppal Road, Tarnaka, Hyderabad 500007, Telangana State, India; ramesh.kandimalla@iict.res.in; 5 Department of Biochemistry, Kakatiya Medical College, Warangal 506007, Telangana State, India

**Keywords:** SARS-CoV-2, COVID-19, ACE2, hydroxychloroquine, cardiovascular system, cardiovascular disease (CVD), therapeutics

## Abstract

The rapidly evolving coronavirus disease 2019 (COVID-19, caused by severe acute respiratory syndrome coronavirus 2- SARS-CoV-2), has greatly burdened the global healthcare system and led it into crisis in several countries. Lack of targeted therapeutics led to the idea of repurposing broad-spectrum drugs for viral intervention. In vitro analyses of hydroxychloroquine (HCQ)’s anecdotal benefits prompted its widespread clinical repurposing globally. Reports of emerging cardiovascular complications due to its clinical prescription are revealing the crucial role of angiotensin-converting enzyme 2 (ACE2), which serves as a target receptor for SARS-CoV-2. In the present settings, a clear understanding of these targets, their functional aspects and physiological impact on cardiovascular function are critical. In an up-to-date format, we shed light on HCQ’s anecdotal function in stalling SARS-CoV-2 replication and immunomodulatory activities. While starting with the crucial role of ACE2, we here discuss the impact of HCQ on systemic cardiovascular function, its associated risks, and the scope of HCQ-based regimes in current clinical settings. Citing the extent of HCQ efficacy, the key considerations and recommendations for the use of HCQ in clinics are further discussed. Taken together, this review provides crucial insights into the role of ACE2 in SARS-CoV-2-led cardiovascular activity, and concurrently assesses the efficacy of HCQ in contemporary clinical settings.

## 1. Introduction

A new type of pneumonia outbreak surfaced in December 2019 in Wuhan, Hubei province, China, which was caused by a novel coronavirus, *viz.,* severe acute respiratory syndrome coronavirus (SARS-CoV)-2 [1]. The pandemic disease, named coronavirus disease 2019 (COVID-19), had 5,867,727 confirmed cases by 29th May 2020 and resulted in 362,238 deaths globally, as sourced by the Coronavirus Resource Center, John Hopkins University (JHU) (https://coronavirus.jhu.edu/). SARS-CoV-2 shares 82% genomic similarity with the other SARS-CoVs, while two other bat-SARS-CoV-like viruses (retrieved from Rhinolophus sinicus, Zhoushan, China), *viz.,* bat-SL-CoVZC45 and bat-SL-CoVZXC21 were found to have >89% similarity [1]. To date, SARS-CoV-2 has crossed all continental boundaries, and presently Europe and North America have been its major epicenters. The COVID-19 symptoms are comparable to those produced by SARS-CoV and Middle East respiratory syndrome (MERS). However, the earliest estimate showed its lower (2%) fatality rate, while about ~ 20% of COVID-19 patients had developed severe conditions [2]. SARS-CoV-2 tropism to the lungs/respiratory system is prominent, in which it infects the lung cells and causes interstitial pneumonitis that may lead to developing acute respiratory distress syndrome (ARDS) and manifestations related to the cardiovascular (CV) system causing multiple organ failure [3,4,5,6,7,8]. Amongst severe COVID-19 patients, 23% of cases had cardiac injuries [9] and, therefore, highlighted this as a common feature that promotes disease severity. Of note, elevated levels of creatinine kinase (CK; >200U/L) in 13% of COVID-19 patients in the general cohort, where most of these lacked any cytokine storm-induced systemic inflammatory response, further affirmed the association of COVID-19 with cardiovascular complications [2]. The common CV complications reported in COVID-19 patients include arrhythmia, myocardial injury (marked by higher troponin I (hs-cTnI) and CK levels) and myocarditis, acute myocardial infarction, acute heart failure and cardiomyopathy, and disseminated intravascular coagulation (DIC) [3,4,10,11]. Although the association of SARS-CoV-2 infection with these manifestations is now known, preexisting CV comorbidities could further contribute to COVID-19 severity and mortality [3,4,10,11]. The earliest report describing a meta-analysis of the COVID-19 clinical cohort revealed a strikingly high existing prevalence of hypertension and cardiovascular disease (CVD) in hospitalized patients, that made them prone to require critical care [10]. COVID-19 patients with CVDs were found to have a relatively five-fold higher mortality risk as compared to the patients with no CVD background [4].

SARS-CoV-2 interacts with an ACE (Angiotensin-converting enzyme) homolog, *viz.,* transmembrane angiotensin-converting enzyme 2 (ACE2) to enter border-line host cells including type II pneumocytes, perivascular pericytes, macrophages, and cardiac cardiomyocytes [12,13]. ACE2 is a carboxy-monopeptidase and an essential component of the renin-angiotensin system (RAS), where it critically participates in maintaining normal CV functions while its dysregulation, observed in multiple CVDs, includes hypertension, myocarditis, and heart failure [14]. Expression of ACE2 on pericytes and cardiomyocytes brought heart and CV tissues to potential risk for SARS-CoV-2 infection, and therefore explained a higher prevalence of CV complications in COVID-19 patients. With the evolving COVID-19 pandemic situation, tremendous pressure and a lack of targeted anti-viral or vaccine prompted researchers and clinicians to consider all available therapeutic options. In this context, the two aminoquinolines, *viz.,* Chloroquine (CQ) and Hydroxychloroquine (HCQ, a less-toxic derivative of CQ) were repurposed widely as therapeutic options for COVID-19. In multiple reports earlier, CQ was shown to be effective in inhibiting SARS-CoV viral replication in vitro [15,16,17]. This evidence prompted an early assessment of CQ and HCQ efficacies against SARS-CoV-2 [18,19,20], where in post-SARS-CoV-2 infection HCQ was found to impair viral replication more effectively than CQ [18]. These preliminary in vitro findings pave the way to assess the therapeutic application of HCQ in clinical studies [21,22,23,24,25]. As of May 29, 2020, searching with “COVID” and “Hydroxychloroquine” terms, 206 clinical trials including that of the National Institutes of Health (NIH) are in progress to assess the therapeutic utility of HCQ globally (details available at https://clinicaltrials.gov/ct2/home). HCQ’s anecdotal repurposing is now being extensively exercised in clinics worldwide. However, in the light of SARS-CoV-2 infection, ACE2 function, and emerging CV challenges, we lacked a clear understanding of HCQ’s pharmacology, mode of action, benefits, and inevitable risks for COVID-19 patients. In this review, we provide insights into the crucial part ACE2 plays in SARS-CoV-2 infection and its significance in systemic cardiovascular function and reviewed the impact of HCQ on SARS-CoV-2 replication and immunomodulatory activities. Taking readouts from clinical COVID-19 studies so far, we reviewed cardiovascular risk and the benefits of HCQ in current clinical settings. We further brief on key considerations in HCQ repurposing and its future perspectives.

## 2. SARS-CoV-2, ACE2, and Cardiovascular Challenges

SARS-CoV-2 is a non-segmented, single-stranded (ss), positive (+) sense RNA virus [26]. It belongs to the family of enveloped RNA beta-coronavirus. Out of seven known species of beta-coronavirus, only three (SARS, MERS, and COVID-19) cause potentially fatal human disease. SARS-CoV-2 produces a 50–200 nanometers virion that is constituted by four structural proteins, *viz.,* the S (spike), E (envelope), M (membrane), and N (nucleocapsid), wherein the N protein is aligned with its RNA genome, while the S, M, and E proteins collectively constitute the viral envelope [27]. The S protein at the SARS-CoV-2 envelop resembles a spike projection that serves as a tool for it to enter the host cell [28]. Phylogenetic analysis revealed 99% similarity of S protein comparing SARS-CoV-2 and SARS-CoV [29] and therefore reaffirmed the evidence that SARS-CoV-2 exploits the same ACE2 receptor [1] that originally served as a functional receptor for SARS-CoV [30].

**Figure 1:**
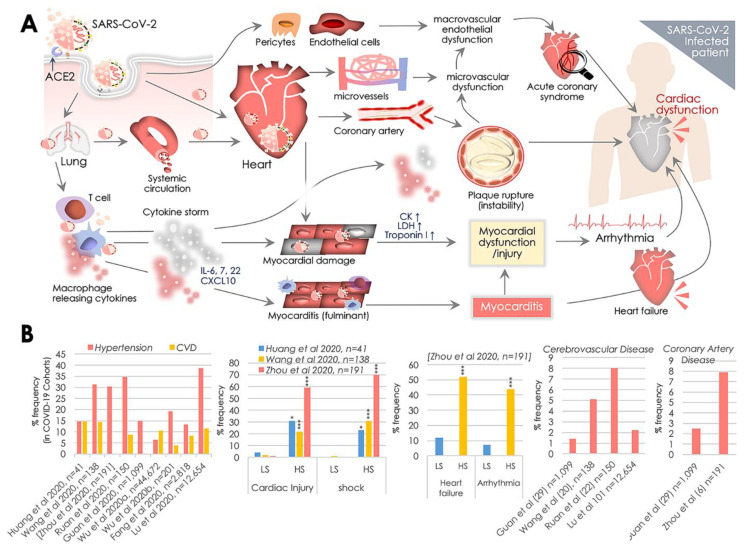
SARS-CoV-2, angiotensin converting enzyme 2 (ACE2), and cardiovascular complications. (**A**) Transmembrane ACE2 receptor facilitates SARS-CoV-2 entry to host cell primarily in the lungs, and then the vascular system, postulating cardiovascular complications by causing inflammation and myocardial dysfunction. SARS-CoV-2 access to the systemic circulation via the lungs potentiates heart infection, while its direct infection of associated pericytes and endothelial cells may cause vascular endothelial dysfunction. Cardiac SARS-CoV-2 infection causes micro-vessel dysfunction, and elevated immunoreactivity disrupts atherosclerotic plaques leading to the progression of the acute coronary syndromes. SARS-CoV-2 infection of alveolar pneumocytes (type II) cells progressively develops the systemic inflammation and elevated immunoreactivity that eventually produces the ‘cytokine storm’, marked by elevated IL-6, IL-7, IL-22, and CXCL10 cytokine levels. It potentiates T-cell and macrophage activation infiltrating infected myocardial tissues and may produce severe cardiac damage and myocarditis, leading to heart failure. Cytokine storm may further increase damage of cardiac monocytes causing myocardial dysfunction and subsequent development of arrhythmia. These events cumulatively produce cardiac dysfunction. (**B**) Manifestation (%) of cardiovascular complications in hospitalized COVID-19 patients reported in key clinical studies exhibiting comorbidities including hypertension, cardiovascular disease (CVD), cerebrovascular disease, coronary artery disease and rate of cardiac injury, shock, heart failure, and arrhythmia in low (LS), and high severity (HS) patient groups. *p* values indicate *** (<0.001), ** (<0.01), and * (<0.05) statistical significance.

ACE2 is present in alveolar epithelial cells and frequently localized at the cell membrane of enterocytes (intestine), pericytes, cardiomyocytes, and macrophages [12,13,31]. ACE2 at the surface of pericytes and cardiomyocytes serves a vital activity of the RAS by maintaining normal CV functions by catalyzing the Ang (angiotensin) I and II [14]. SARS-CoV-2′s S protein primarily binds to the ACE2 of alveolar epithelial cells in the respiratory tissues that enable its further access to the systemic circulation, reaching cardiomyocytes in the heart and pericytes and endothelial cells in the macro-vessels (Figure 1A). Endocytosis-driven internalization of ACE2 on the membrane of cardiomyocytes, pericytes, and endothelial cells by SARS-CoV-2 results in omitting ACE2 from the cell surface and potentially raises the risk of CV complications in COVID-19 patients [32]. The loss of ACE2 carboxypeptidase function was earlier shown to compromise cardiac function [33]. A higher ACE2 level in patients with existing CVD and/or hypertension was also suggested to increase the susceptibility to SARS-CoV-2 infection [34]. In light of this information, clinical readouts from six studies, including 1527 COVID-19 patients, revealed 17.1%, 16.4%, and 9.7% prevalence of hypertension, cardiac & cerebrovascular disease, and diabetes, respectively [10]. Prevalence of these CVD comorbidities was found to be higher in patients requiring ICU than the non-ICU patient groups. Analyses of mortalities in a cohort of 44,672 COVID-19 patients from Wuhan, China also showed 10.5%, 7.3%, and 6% mortalities in patients having CVD, diabetes, and hypertension, respectively, significantly greater than the overall mortality rate (2.3%) for COVID-19 patients [4]. To date, nine clinical studies from China [2,4,5,6,7,8,35,36,37] have comprehensively assessed CV comorbidities in COVID-19 patient cohorts and yielded similar clinical results (Figure 1B). However, disparities in testing, standardization and options for standard procedure in clinical studies from China [6,8,38,39] [2,37,40] and elsewhere [40] have impacted the quantitative clinical outcomes. To assess the cardiovascular outcomes of SARS-CoV-2 infection in a recent report, Liu et al. reported a significantly higher level of circulating Ang II in COVID-19 patients than the controls; circulating Ang II in levels COVID-19 patients also correlated well with viral load [41]. Of note, these results were consistent with reduced ACE2 activity. They again underlined the crucial role of RAS in COVID-19 disease and reaffirmed the focus on the cardio-protective function of ACE2, where an alteration in its activity may substantially impact the cardiovascular outcomes [33,34]. Therefore, in light of these reports, ACE2 has gained recognition as a key and central target in COVID-19 pathology and associated CV complications. Taking note of SARS-CoV-2 infection severity, here we review the frequent clinical cardiovascular complications observed in COVID-19 patients and further shed light on the potential involvement of ACE2 activity.

### 2.1. Myocardial Injury, Shock, and Congestive Cardiac Failure

An earlier diagnostic assessment of COVID-19 patients in Wuhan revealed an increase in the levels of cardiac cTnI (a myocardial injury marker >28 pg/mL) in five of the first 41 patients [6,34]. While recent analyses exhibited 7.2% [8] to 17% [7] incidences of acute myocardial injury in hospitalized COVID-19 patients, such risk was found to be six to twelve-fold higher in the high severity (HS) group than the low severity (LS) patient group (Figure 1B). Of note, multiple reports showed that elevated levels of lactate dehydrogenase (LDH) and serum creatine kinase (CK) were evident in almost all hospitalized COVID-19 patients [6,34,42]. Cases of other cardiac complications such as fulminant myocarditis were also evident and were suggested to be the outcome of SARS-CoV-2 infection (Figure 1A). A recent study reported 23% cases of congestive cardiac failure in COVID-19 patients among all in-hospital Chinese patients (Figure 1B). Markedly, these incidences in deceased and survivor cases were found to be 52% and 12%, respectively [7]. A lack of information on the possible association of cTnl increase with pre-existing CV complications limits our ability to predict causalities. However, an increase in cTnl levels was found to depict poor prognosis in other systemic diseases. Therefore, such associations of cTnl levels with prognosis is likely to predict the risk of systemic diseases, e.g., hypotension or hypoxia, then a specific cardiac dysfunction. In the given context, the role of the ‘cytokine storm’ elicited by SARS-CoV-2 immunoreactivity appears to be a key mediator [34]. Aberrant expressions of a variety of cytokines are evident in severely ill COVID-19 patients, while elevated plasma interleukin-6 (IL-6) levels were seen in patients with cardiac injury [43] (Figure 1A). Given the fact that ACE2 levels are present at the cell surface and in circulation in the CV system, direct SARS-CoV-2′s cardiomyocyte infection is suggested to be a definite possibility [44].

### 2.2. Cardiac Arrhythmia

Manifestations of viral infections are frequently seen to be associated with myocardial inflammation, metabolic dysfunction, and modulation of the sympathetic nervous system that all serve as key factors in causing the cardiac arrhythmia. A meta-analysis of 138 COVID-19 patients cohort reported 16.7% incidences of developing arrhythmia in patients, which in terms of serious complications came second after ARDS [8]. Another cohort comparing patients based on their admittance to ICU revealed strikingly higher (44%) cases of arrhythmia in ICU admitted patients, while in the non-ICU admitted patients group it remained at 4% [39] (Figure 1B). These findings postulated the role of systemic inflammation and elevated immunoreactivity produced by cytokine storm that may damage cardiac monocytes causing myocardial dysfunction and subsequent development of arrhythmia (Figure 1A). The internalization of ACE2 by SARS-CoV-2 served as a key event that led to the altered RAS system, which was postulated earlier to cause pro-inflammatory and pro-oxidant activities [32].

### 2.3. Myocarditis

Acute viral infections are known to cause cardiac injury and acute myocarditis. A recent report by the National Health Commission, China, showed the infiltration of mononuclear cells and the onset of monocyte necrosis in cardiac muscle autopsy specimens. Along these lines, other findings concerning fulminant myocarditis indicate the possibility of myocarditis in COVID-19 patients as a cause of acute cardiac injury [43,45]. Despite the findings of these reports and individual clinical cases [43,45], we presently lack any information on the underlying mechanism, its prevalence, and clinical importance, and therefore this emphasizes the need for detailed clinical analyses. However, the earliest reports suggested that fulminant myocarditis may potentially be a clinical manifestation of SARS-CoV-2 infections of cardiomyocytes [43,45], postulated to be caused by elevated IL-6, IL-7, IL-22, and CXCL10 cytokine levels produced as a result of cytokine storm (Figure 1A). ACE2-led SARS-CoV-2 infection of alveolar pneumocytes (type II) cells has been suggested to trigger the onset of systemic inflammation and elevated immunoreactivity leading to a ‘cytokine storm’, that may essentially potentiate T-cell and macrophage activation infiltrating infected myocardial tissues and resulting in cardiac damage and myocarditis (Figure 1A). However, a detailed assessment of these events is needed to further confirm the acquisition of systemic myocarditis in COVID-19 patients.

### 2.4. Acute Coronary Disease (ACD) and Ischemia

Most clinical studies so far lack any insights into ACD in COVID-19 patients; however, it is suggested that it impacts on destabilizing coronary plaques in COVID-19 patients [7,46,47]. Of note, the role of the systemic inflammatory response is implicated primarily in destabilizing atherosclerotic plaques [48], which further supports pro-inflammatory and pro-oxidative consequences of SARS-CoV-2-led ACE2 loss in COVID-19 patients (Figure 1A,B). More specifically, COVID-19 patients with heart failure are at higher risk of acute events or ischemic syndrome.

### 2.5. Disseminated Intravascular Coagulation (DIC)

Incidences of pulmonary embolism (PE) and subsequent disseminated intravascular coagulation (DIC) are linked with coronavirus infection, as COVID-19 patients demonstrate a hypercoagulable state, marked by prolonged prothrombin time, elevated D-dimer level and fibrin split. Of note, 71.4% of non-survivor patients were found to have DIC [49]. COVID-19 patients characteristically also had vast pulmonary embolism features [50]. Importantly, increase in D-dimer in COVID-19 patients was suggested to predict adverse survival outcome, for instance, a study of a retrospective cohort showed that increased D-dimer levels (>1 g/L) were able to closely predict in-hospital mortality [7]. However, the mechanistic basis of these features of SARS-CoV-2 infection is yet to be elucidated, while new knowledge of pro-inflammatory/oxidant activities in these syndromes could further shed light on the underlying role of ACE2 function in SARS-CoV-2 pathogenesis.

### 2.6. Immune Function in Cardiovascular Complications

After respiratory infection, the immune response is the second most exploited system in COVID-19 patients, and this has severe implications for the cardiovascular system. Firstly, Huang et al. reported elevated systemic IL-2, IL-6, IL-7, C-X-C motif chemokine 10 (CXCL10), chemokine (C-C motif) ligand 2 (CCL2), tumor necrosis factor-α (TNFα), and granulocyte colony-stimulating factor (G-CSF) levels in COVID-19 patients [6]. The elevated levels of systemic cytokines shared clinical features with cytokine release syndrome (CRS) [13] that may substantially contribute to COVID-19 severity. The above systemic immune response resembles cytokine profiles raised in hemophagocytic lympho-histiocytosis (HLH) syndromes [51]. Sorting of the immune cell population in COVID-19 patients revealed the presence of hyperactivated T-cells with high fractions of HLA-DR+, CCR6+ Th17 CD4+ and CD38+ CD8+/CD4+ T-cells. This emphasized the role of hyperactivated T-cells, which may partly be associated with severe immune injury [13]. Furthermore, elevated levels of circulating IL-6 in a cohort of 150 patients in a recent retrospective study were found to be predictive of mortality in hospitalized COVID-19 patients [5]. Of note, the role of IL-6 has been earlier primarily implicated in CV complications, including atherosclerosis and coronary heart disease, and with increasing the risk of cardiac inflammation and morbidity [52,53]. Therefore, the prevalence of systemic cytokine response/CRS or cytokine storm in clinical COVID-19 patients significantly raises an obvious risk of cardiovascular complications (Figure 1A).

## 3. ACE2 Receptor and Its Significance in Systemic Cardiovascular Function

**Figure 2:**
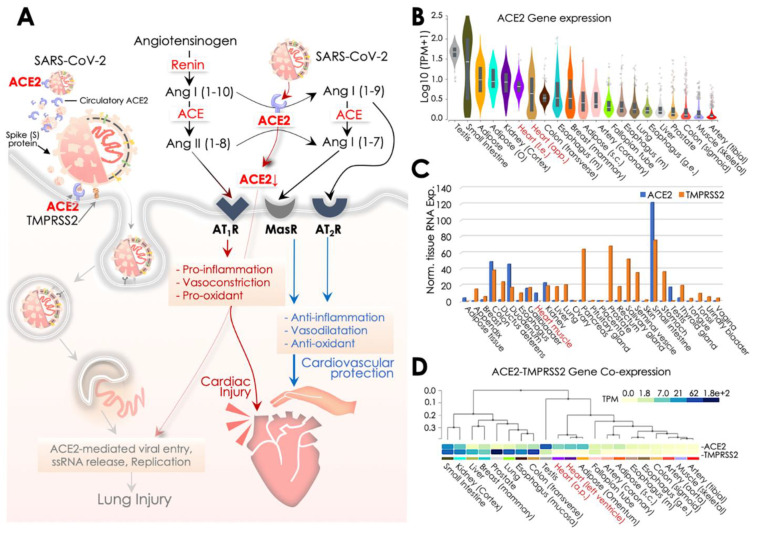
SARS-CoV-2 pathology and ACE2-led regulation of cardiovascular function of the Renin-Angiotensin System (RAS). (**A**) Schematic diagram illustrating the central role of ACE2 in SARS-CoV-2 recognition and the differential regulation of the RAS system for cardiovascular protection or cardiac injury. SARS-CoV-2 spike (S) protein undergoes priming by the TMPRSS2, a host cell membrane protease, and it subsequently binds to ACE2 infecting the host cell. In the RAS system, ACE2 activity with MasR, and AT2R receptors provides cardiovascular protection. In contrast, a reduced ACE2 activity as a result of its binding to SARS-CoV-2 and engulfment into the cell may elevate ACE activity and Ang II levels that essentially potentiates cardiac damage/injury. (**B**) ACE2 gene expression data of ACE2 retrieved from Genotype-Tissue Expression (GTEx) showing its expression across human tissues, wherein heart tissues are marked in red at x-axis. Expression values are shown in the log10 scale for TPM (Transcripts Per Million) unit. (**C**,**D**) ACE2 and TMPRSS2 mRNA levels retrieved from Human Protein Atlas (HPA; C) and Genotype-Tissue Expression (GTEx; D) showing their co-expression across various human tissues; heart tissues are marked in red at x-axis.

ACE2 comprises an 805-amino acid (aa; Mr 110,000 glycoprotein) long endothelium-bound carboxy-mono-peptidase that consists of a 17-aa N-terminal peptide (catalytic domain-oriented extracellularly) and a C-terminal anchor integrated into the membrane. ACE2 is catalytically a zinc metalloprotease and the only homolog of ACE known in humans [54]. ACE2 is part of the RAS that plays a crucial function in maintaining normal cardiovascular functions, while dysfunction in RAS contributes to CVDs, including hypertension, myocarditis, coronary heart disease, and heart failure [14]. RAS is constituted by a set of catalytic enzymes that includes angiotensinogen, renin, Ang II, Ang II receptors (AT1R and AT2R), and ACE [55]. Among these, ACE2 has a crucial role to play by catalyzing Ang II to Ang (1–7) or Ang I to Ang (1–9) [56]. ACE2 can access substrate/peptide in the circulation, and it is known for its circulatory presence and catalytic function in the blood and body fluid. Given its carboxy-monopeptidase activity, ACE2 primarily trims the COOH-terminal phenylalanine residue from Ang II [57]. ACE2-led trimming of Ang II to Ang (1–7) is a significant event in the RAS, since the role of Ang II is critically implicated in producing hypertension by promoting vasoconstriction, fibrosis, Na+ retention, and pro-inflammation and pro-oxidant activities. At the same time, elevated levels of Ang (1–7) peptide inhibits the Ang II/AT1R axis and induces anti-inflammatory, anti-oxidant, anti-fibrotic, and vasodilatory activities (Figure 2A) [56,58]. Therefore, ACE2 activity switches on the processing of Ang II in the classical RAS system and loss of ACE2 or its function could put the RAS system to an overall higher Ang II level [58].These cardioprotective activities of ACE2 are regulated through the Ang I (1–9)/AT2R and Ang I (1–7)/MasR axes [55].On the contrary, ACE degrades Ang (1–7) and forms ANG II that results in promoting inflammation, fibrosis, and high blood pressure (Figure 2). The role of ACE2 was also implicated in the hydrolysis of apelin and des-arginine bradykinin (des-Arg1-BK) apelin peptides, wherein des-Arg1-BK was shown to have a pro-inflammatory function via stimulating the B1 receptor [59] (Figure 2). Besides its critical role in the CV system, ACE2 was earlier discovered to be a key binding receptor for SARS-CoV and NL63 (HCoVNL63) coronaviruses [30,60], while recently it was identified to be a SARS-CoV-2 receptor [61]. ACE2 is also shown to play a key role in acute respiratory/lung injury caused by influenza viruses *viz.*, H1N1, H5N1, and H7N9 [62,63,64].

Mechanistically, the intracellular entry of SARS-CoV-2 in host cells is facilitated by binding of its S (spike) protein’s receptor binding region with the ACE2 extracellular domain, at a high affinity (15 nM) [65]. Prior to their binding, the host cell serine protease, *viz.,* TMPRSS2S, processes cleavage of S protein down the dibasic Arg sites and yields to the S1 and S2 subunits. S protein cleavage is a crucial step which enables S2-led membrane fusion and ACE2-mediated SARS-CoV-2 internalization by endocytosis [66,67] in the type II pneumocytes, pericytes, or cardiomyocytes [12,13]. Structural analyses suggest that S protein of SARS-CoV-2 has a receptor-binding domain (RBD) to interact with human ACE2, wherein 441Leu, 472Phe, 479Gln, 480Ser, 487Asn, and 491Tyr residues of S protein were predicted to have a critical role in its binding [68]. Higher virulence of SARS-CoV-2 than SARS-CoV was shown to reflect a higher affinity of S1 protein for ACE2 [69]. SARS-CoV-2 binding of ACE2 at the membrane and its subsequent loss by endocytosis impact RAS and change the overall Ang II:Ang (1–7) ratio (enriching cardio-inflammatory Ang II, and decreasing cardio-protective Ang (1-7) levels), eventually exacerbating cardiac injury by SARS-CoV-2. However, the extent of CV tissue damage due to the presence of SARS-CoV-2 in the circulation has not been precisely analyzed as yet [6,70]. ACE2 is a primary established route of Ang II metabolism that generates Ang (1–7) in the heart, and therefore its loss is frequently seen as compromising systemic cardiovascular function [32,71,72,73], wherein hypertension, inflammation, vasoconstriction, and oxidative activities have been the common CV complications [32,60,74,75].

Given the fact that ACE2 circulating levels are endogenously deficient, its adequacy in preventing viral dissemination by sequestering SARS-CoV-2 in circulation was questioned [54], yet its protective effects against hypertension, myocardial hypertrophy, inflammation, and fibrosis were evident [76]. Also, amid these speculations [54,77], a proposed clinical trial (NCT04287686) to study the infusion of recombinant ACE2 to restrict viral infection was subsequently withdrawn. However, a recent report testing the potential of clinical-grade human recombinant soluble ACE2 (hrsACE2) in engineered human tissues showed an effective SARS-CoV-2 inhibition by a factor of 1000–5000. Therefore, soluble ACE2 might be the key therapeutic alternative restricting SARS-CoV-2 infection at early stage [78]. An alternative strategy using ACE2-specific antibodies to target membrane-aligned and soluble ACE2 was earlier found to be effective against SARS-CoV infection [79]. ACE2, inhibition of TMPRSS2 in the murine models also limited coronavirus infection and improved survival [80,81]. Presently, two ongoing clinical trials (NCT04321096 & NCT04338906) are testing the efficacy of TMPRSS2 inhibition by camostat mesilate to evaluate its benefits against COVID-19.

TMPRSS2 is critical in the host recognition of SARS-CoV-2, and therefore for the S protein it serves as a cofactor for ACE2-mediated viral entry into the host cell [12]. Hence, TMPRSS2 and ACE2 co-expression is crucial to facilitate an optimum SARS-CoV-2 infection. To get an insight into the above possibility, we firstly surveyed the ACE2 transcript expression at Genotype-Tissue Expression (GTEx) that revealed its enriched expression, particularly in testis, small intestine, adipose and heart/cardiac tissues (Figure 2B). This primarily explains the vulnerability of these tissues to SARS-CoV-2 infection. Of note, ACE2 transcript levels in the heart were higher than in the lungs which could help to explain the higher vulnerability of SARS-CoV-2 infection in the CV system and the prevalence of CV complications in COVID-19 patients. Similar observations were obtained in a recent report [44]. However, our survey of ACE2 and TMPRSS2 transcript co-expression at Human Protein Atlas (HPA; http://www.proteinatlas.org) further revealed a lesser co-occurrence of their expressions in the heart, while a strong correlation of their co-expression was observed in intestinal (colon, duodenum, small intestine) and renal tissues [82] (Figure 2C). Of note, a moderate expression of ACE2 and its co-expression with TMPRSS2 in the kidney/renal tissues also underlines association of SARS-CoV-2 infection with renal injury, which is frequently seen in COVID-19 patients. In critically ill patients admitted to ICU, 0.5–29% incidences of acute kidney injury have been reported [2,6,7,8,9,38], and, thus, kidney injury was also recognized as a key feature of disease severity and correlated negatively for patient survival [7]. Interestingly, enzymatically active/secretory tissues (pancreas, prostate, salivary gland, seminal vesicle, stomach, and thyroid) were more enriched with TMPRSS2 expression. A much similar co-expression pattern of ACE2 and TMPRSS2 transcript was observed in the GTEx database (Figure 2D). Of note, in both analyses, a higher expression of TMPRSS2 was more evident in the lungs than the heart. However, the functional significance of such disparity in its expression warrants further investigation. Results of these surveys overlapped in parts with findings of an earlier report that analyzed co-expression of ACE2 and TMPRSS2, along with HAT in influenza and SARS-coronavirus, and reported their evident co-expression in respiratory, gastro-intestinal and cardiovascular tissues [83]. This evidence postulates that ACE2 and concomitant TMPRSS2 expression in pericytes and cardiomyocytes could essentially potentiate chances of SARS-CoV-2 infection in the systemic CV system and also explain the higher prevalence of CV issues in COVID-19 patients.

## 4. SARS-CoV-2, ACE2, Hydroxychloroquine and beyond: Preventive and Therapeutic Aspects

The ongoing COVID-19 pandemic prompted the urgent need to develop targeted therapeutic strategies and exercise all options of repurposing conventional drugs as a viable solution, considering their known pharmacological aspects and benefits. Efforts identified CQ and HCQ as two therapeutic drugs potentially useful in preventing COVID-19. The earliest reports analyzing the effects of CQ and HCQ in vitro against SARS-CoV-2 revealed their inhibitory antiviral activities [19,84,85]. Besides their earlier known antimalarial activity [86], these aminoquinoline analogs provide broad-spectrum benefits against types of bacterial, fungal, and viral infections [87,88,89]. Given its inexpensive cost, less toxicity, good tolerance, and immunomodulatory activities in patients [90], early reports in the last decade explored the repurposing of CQ against human immunodeficiency virus (HIV) and other viruses causing inflammation [91]. Therapeutic benefits of CQ’s anti-inflammation and immunomodulatory activities were also explored in autoimmune diseases [92].

Keyaerts et al. in 2004, first demonstrated the antiviral activity of CQ against SARS coronavirus [16], while further reports revealed an inhibitory function of CQ on HCoV- 229E replication in epithelial lung cell in vitro [93,94]. Keyaerts et al. in 2009 also showed that infection of HCoV-O43 coronavirus in newborn mice could be treated by medication of CQ through the mother’s milk [95]. With growing concerns about the toxicity of CQ medication in humans, HCQ, a less toxic derivative of CQ quinine analog, was subsequently opted for and used in clinical studies broadly. While keeping SARS-CoV-2 and ACE2 in focus, in the following sections we discuss the pharmacology of HCQ, its benefits in vitro and clinical settings, and what impact HCQ has upon SARS-CoV-2 replication and underlying immunomodulatory activities.

### 4.1. HCQ Pharmacology, In Vitro and Clinical Outcomes

The HCQ parental molecule, i.e., quinine, was first extracted from the Cinchona, a native tree to Peru, and primarily used for its benefits against malaria [96]. Later, an amine acidotrophic form of quinine, *viz.,* CQ, was synthesized in Germany in 1934 as a natural substitute. CQ and its 4-aminoquinoline derivative, *viz.,* HCQ, are both weak bases and share a common molecular family. Addition of a hydroxyl group at the CQ side-chain terminal end (β- hydroxylation of the N-ethyl substituent) forms HCQ. It is usually administered orally in the form of HCQ sulfate, while its pharmacokinetics are similar to those of CQ, which include its swift absorption in the gastro-intestine and its renal release. HCQ, being a positively charged base, stays in a protonated form. However, un-protonated HCQ can access the intracellular organelles/compartments, where it becomes protonated, which, in turn, increases the localized pH. This mechanism explains the accumulation of CQ/HCQ within acidic organelles, e.g., endosome, lysosomes, and the Golgi vesicles [97], and thus becomes one aspect of its pharmacokinetic activity. This fact may also explain its 200–700-times higher accumulation in splenic, hepatic, renal, and heart and lung than in plasma [98]. With its quick absorption, HCQ achieves its maximum concentration in serum in 2–3.5 h, while the half-life of its clearance was 22–45 days [99].

Multiple in vitro studies performed earlier on SARS-CoV, exhibiting the benefits of CQ or HCQ against viral replication, provided an early hope for the potential repurposing of CQ/HCQ against COVID-19. Firstly, Keyaerts et al. in 2004 demonstrated that sub-toxic CQ concentration (8.8 +/− 1.2 μM, much lower than the CC50 (261.3 +/− 14.5 μM)) could effectively reduce the SARS-CoV replication rate in Vero E6 (kidney epithelial; source-African green monkey) cells by 50% [16]. In another report, Vincent et al. showed that, at 10μM, CQ concentration effectively inhibited SARS-CoV viral replication in Vero E6 [15]. These inhibitory effects of CQ treatment were effective in pre- or post-SARS-CoV infected cells and therefore hinted at its prophylactic and therapeutic applications [15]. Biot et al. also observed a similar finding with CQ and HCQ; however, they reported that CQ causes more potent inhibition of viral replication [17]. These reports pitched the anecdotal benefits of CQ/HCQ for their repurposing against the ongoing COVID-19 pandemic and prompted researchers to evaluate their activity against SARS-CoV-2. In the earliest efforts, Yao et al. analyzed the antiviral activities of HCQ and CQ on SARS-CoV-2 in Vero cell lines [18]. HCQ and CQ both showed SARS-CoV-2 inhibitory activity; however, in contrast to the study by Biot et al., which show better efficacy of CQ against SARS-CoV [17], Yao et al. showed that HCQ (EC50 = 0.72 μM) has much higher antiviral potency than CQ (EC50 = 5.47 μM) for SARS-CoV-2 [18]. In a post-infection treatment regime, HC impaired viral replication more effectively. While analyses of prophylactic activity also indicated the greater efficacy of HCQ (EC50-5.85Μm) than CQ (EC50-18.01Μm) in 48 h, an extended treatment suggested the production of a more significant anti-viral effect [18]. To determine a potential clinical regime for HCQ, Yao et al. enrolled physiology-based pharmacokinetic modeling and considered multiple parameters, including drug administration route, its physiological assimilation (i.e., intestinal absorption and accessibility to lung tissue), and biochemical activities. This report also discussed simulated concentrations of lung fluid but lacked inclusion of all details used in the model [18]. Based on physiology-based pharmacokinetic modeling, Yao et al. proposed a treatment regime that included an initial dose of 400 mg HCQ twice a day and a continuation of 200 mg dose twice daily for the next four days, which turned out to be a key outcome of this study. However, the study lacked a 95% confidence interval value for the estimated EC50 dose, which suggests that the aforementioned dose regime needs to be adopted with caution to avoid inaccuracy in treatments [18]. Another report by Liu et al., using a similar antiviral regime at four multiplicities of infection, revealed the efficacy of CQ and HCQ in inhibiting SARS-CoV-2 viral replication at all four tested infection regimes [85]. Although they suggested a more robust potency of CQ than HCQ, this was only found to be significant at 0.01 and 0.2 multiplicities of infection. Importantly, using immunofluorescence-based co-localization assay, they analyzed entry of SARS-CoV-2 virion into the endosome-lysosome proteolysis pathway and found an accumulation of more virions at early endosomes and lesser at endolysosomes in CQ and HCQ treated cells in comparison to untreated viral infected control cells [85]. Using a similar Vero E6 cell system, Wang et al. further showed that a combination of remdesivir (EC50 = 0.77 μM) and CQ (EC50 = 1.13 μM) could effectively control viral infection in vitro [19]. This study enrolled SARS-CoV-2 at a multiplicity of infection (MOI) of 0.05 and pre-treated Vero E6 cells with 0.01, 0.05, 0.1, 0.5, 1, 5, and 10 μM CQ for 1 h. Regarding the antiviral activities of remdesivir and CQ, Wang et al. suggested their inclusion in clinical therapeutic regimes against SARS-CoV-2 [19]. In another combinatorial approach, Andreani et al. showed a synergistic effect of CQ and Azithromycin (AZM) against SARS-CoV-2 [100]. Using concentrations of 1, 2 or 5 μM CQ along with 5 or 10 μM AZM and multiplicity of infection (MOI) at 0.25 they showed that 5 μM CQ treatment in combination with 10 and 5 μM AZ led relatively to 97.5% and 99.1% viral inhibition respectively [100]. The details of these in vitro studies testing efficacies of CQ and HCQ against SARS-CoV-2 are provided in Table 1, where the 2004 report of Keyaerts et al. is taken as reference.

Initial findings of HCQ/CQ antiviral activity from in vitro studies [18,19,85,100] raised an early clinical interest in testing the efficacy of HCQ/CQ in the clinic for COVID-19 treatment. An interim analysis from China, including more than 100 COVID-19 patients, showed the superiority of CQ treatment compared to the control group [24]. Although this report provided no details of enrolled patients, their clinical features including the benefits of HQ in improving lung imaging, viral shedding, and in shortening disease course were discussed. One key takeaway from this study was the recommended CQ dose (500 mg twice daily -b.i.d.) for the next ten days for patients exhibiting mild, moderate or severe symptoms [24].

A comprehensive review of available clinical data so far on the prophylactic and therapeutic use of HCQ/CQ against COVID-19 in human cohorts included nine clinical studies and two case series/reports, as summarized in the Table 2. In the earliest report, Chen et al. analyzed the efficacy of HCQ in a small-size (30 inpatients) randomized controlled trial in Shanghai, China [21]. When comparing the clinical outcome of HCQ to the standard of care, they found no statistically significant differences in virus clearance in control (93%, *p* > 0.05) and HCQ (87%) group by day 7. Also, no difference in the clinical symptoms, including the fever, its duration, and any alteration in lung features was observed in 400 mg HCQ treated patients for five days. Although the admitted patients in control and HCQ groups had symptoms for ~ 6 and 7 days respectively, no detail of COVID-19 severity in the enrolled patients was reported. In mid-March 2020, Gautret et al., in an open-label, non-randomized clinical trial, reported that HCQ causes significant viral clearance at day 6 from the nasopharynx of treated patients (60%) as compared to control (15%) [22]. A faster viral clearance in patients who were given HCQ and Azithromycin (AZM) was reported and hinted at the synergistic effect of the two drugs. Given its non-randomized, unequal settings, and exclusion of six patients from analyses [22], the clinical outcome of this study was criticized. Another report from this group with a non-randomized cohort of 80 patients (with ~5 days symptoms) treated with HCQ and AZM revealed that 93% treated patients were negative of SARS-CoV-2 in just 4.5 days of treatment, as validated using reverse transcription-polymerase chain reaction (RT-PCR) of nasopharynx samples [25]. An absence of a comparison arm in this analysis compromised the clinical outcome of the report. Malina et al., another group from France, testing the combination of HCQ and AZM in a prospective, open-label study showed that, out of ten patients, only two exhibited viral load reduction by day six, and they therefore doubted the clinical outcome of studies published by Gautret et al. [22]. In another report from China, Chen et al. using HCQ for a mild symptomatic COVID-19 patient cohort showed faster clearance of cough and fever in HCQ treated patients than control [101]. However, this report had several limitations, including the exclusion of patients for unclear reasons, delivery of antivirals, steroids, and intravenous immunoglobulin as standard therapy and no endpoint details (no information on mortality, viral clearance, and patient discharge). A preprint study from France, comprising retrospective/non-randomized trials of 181 inpatients, examined the efficacy of HCQ (600 mg/day) in 84 patients, while 97 patients were taken as control [102]. Authors found no apparent benefits of HCQ compared to control, and ~ 10% of patients given HCQ were discontinued due to change in their ECG reading. In a recent multi-centric, randomized controlled trial from China, Tang et al., enrolling 75 patients each in HCQ and control (receiving standard of care) groups, showed no significant difference in viral clearance in HCQ (85.4%) and control (81.3%, *p* = 0.341) by day 28 [103]. Testing a 1200 mg HCQ dose for the first three days followed by 800 mg daily dose for the next two-three weeks, this post hoc trial did not support HCQ use for COVID-19 treatment. Recently, in one of the biggest open-label and non-randomized trial studies (comprising 1061 patients) testing HCQ and AZM combination in France, Million et al. showed, in a ten-day regime, good clinical outcome and viral cure observed in 973 HCQ+AZM treated patients (91.7%) [104]. They recommend that prophylactic use of HCQ+AZ is safe and acquires a low fatality rate in patients. In contrast, in a preprint recent retrospective/non-randomized trial of veterans hospitalized in USA that were given HCQ and HCQ+AZM for groups of 97 and 113 patients respectively, Magagnoli et al. showed no evidence that HCQ, either with or without AZM, benefits patients and lessens the risk of mechanical support in treatment [105].

Besides these clinical trials, one case report and one case series also tested the efficacy of HCQ in COVID-19 patients. In the first case report, Mathies et al. showed that, in a 77-year old moderately sick COVID-19 patient with a history of heart transplant, HCQ treatment restricted further deterioration in his clinical condition, and the patient was released from hospital after twelve days with negative viral load [106]. In a self-controlled case series study (presently in preprint), Lane et al. showed that, compared to the control group given either nothing or Sulfasalazine-SSZ, patient groups treated with HCQ, HCQ+AZM, or HCQ+AMX showed no risk of severe illness [107]. However, HCQ combination with AZM was shown to increase the risk of CVD and mortality in patients. The clinical outcome of HCQ efficacy against COVID-19 in these studies has so far mainly remained confusing and inadequate. Therefore, a need for well-designed, structured, randomized controlled trials is critical to precisely assess the benefits of HCQ against COVID-19.

### 4.2. ACE2, Hydroxychloroquine, and SARS-CoV-2 Replication

**Figure 3:**
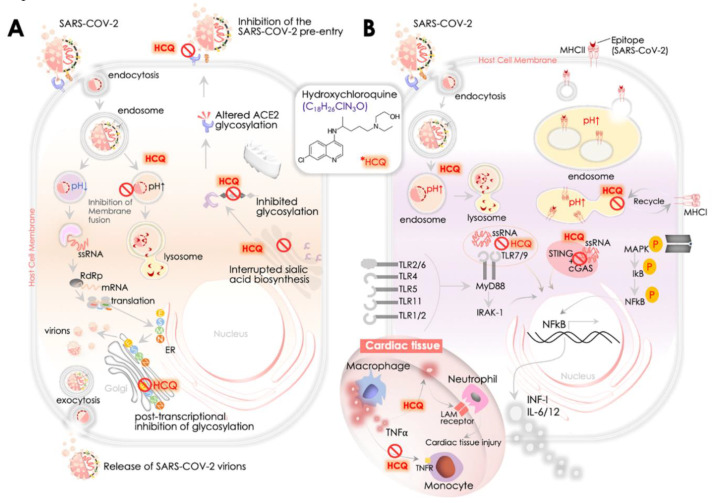
Hydroxychloroquine (HCQ): SARS-CoV-2 replication and immunomodulatory activities -proposed mechanism. (**A**) HCQ impacts the binding of SARS-CoV-2 S-protein and ACE2 receptor at the host cell surface by altering the ACE-2 n-terminal glycosylation. HCQ restricts SARS-CoV-2 infection by increasing endosomal pH that disrupts SARS-CoV-2 envelope fusion (requires acidic pH) with endosome membrane phospholipids and subsequent release of its sRNA genome. This is a crucial step that could intervene in its further replication/transcription by RNA-dependent RNA polymerase (RdRp, *viz.,* nsp12) and synthesis of its spike (S), membrane (M), envelope (E), nucleocapsid (N), and nsp3 (a replicase complex component). SARS-CoV-2 infection exploits host cell’s ribosome machinery to synthesize its non-structural proteins (NSPs) that constitutes a replicase-transcriptase complex that is enrolled further to synthesize its sub-genomic RNA. Viral proteins get translated in ER and processed in Golgi before assembling into the nucleocapsid and budding it as a mature virion. HCQ is postulated to alter the maturation of M protein at Golgi, resulting in the collapse of viral assembly. Besides interrupting glycosylation of the ACE2 receptor, HCQ also seems to restrict biosynthesis of the sialic acids that play a part in host cells binding with SARS-CoV-2. The role of HCQ is also implicated in attenuating the activation of mitogen-activated protein (MAP) kinase that could further impact viral replication. (**B**) HCQ modulates immune function and reduces inflammation. HCQ-led increase in endosomal pH impacts MHC Class I and II antigen cross-presentation. It alters the preparation and development of SARS-CoV-2 Ag-specific T-cells and B-cells. HCQ also impacts the onset of cytokine release from the innate immune system by attenuating DNA/RNA interaction and by activation of cGAS/STING signaling and by disrupting binding to TLR7/9 by increasing the endosomal pH. HCQ impact on these axes further attenuates NFkB nuclear function in promoting the expression of pro-inflammatory cytokine (IFN I, IL-6, IL-12 etc.). In the cardiac tissue, HCQ also attenuates TNFα production in the macrophages and thereby reduces expression of TNFR (TNFα receptor)-1/2 at the membrane of nearby monocytes, which further restricts TNFα’s role in the extravasation of neutrophils that supports opening up the tight junctions of vascular endothelial cells and stimulates leukocyte adhesion molecules (LAM) expression.

The steps of viral entry, replication, and protein synthesis/processing are key druggable targets for antiviral drugs (Figure 3A). In the context of the utility of quinines, Savarino et al. were first to suggest the benefits of HCQ and CQ for the treatment of SARS-CoV [90]. They postulated the involvement of endocytosis in viral entry and associated immune response, where the latter could be a result of the activation inflammatory cytokines contributing further to the severity of viral infection, and therefore hinted at the potential benefits of HCQ and CQ to intervene in the underlying mechanism [90]. An in vitro study by Kayaerts et al. in the subsequent year confirmed the potency of CQ in inhibiting SARS-CoV replication in Vero E6 cells [16], whereas, Vincent et al. showed a dose-dependent inhibition of viral replication in Vero E6 cells, in both cases, either immediate or 3–5 h post-viral infection [15]. Of note, they showed that CQ treated cells had a lesser viral infection, and CQ could impair the terminal glycosylation of the ACE2 receptor, reducing SARS-CoV–ACE2 affinity and eventually diminishing the infection rate. These results emphasized the utility of HCQ for coronavirus prophylaxis [15]. Multiple recent in vitro reports as described in the earlier section [18,19,85,100] further implicated the role of HCQ in the inhibition of SARS-CoV-2 replication. However, we presently lack molecular insights into the mode of action of HCQ/CQ against SARS-CoV-2. Learning from available evidence of its function primarily involves three aspects of its antiviral functions including: (i) inhibition of viral entry by affecting receptor glycosylation, (ii) control of virus replication by abolishing the pH-dependent endosome-mediated viral entry, and (iii) restriction of viral protein’s post-translational modification.

Kwiek and colleagues earlier revealed that QC could attenuate viral infection by interfering with the pre-entry step of viral recognition on the host cell receptor [108] (Figure 3A). Mechanistically, CQ was found to inhibit the function of quinone reductase 2 [108], a close structural relative of the UDP- N -acetylglucosamine 2- epimerases [109] enzyme that plays a critical function in sialic acid biosynthesis. Sialic acids are acidic monosaccharides that are frequently found at the edge of sugar chains of many transmembrane receptors/proteins and facilitate ligand binding. Of note, orthomyxoviruses and human coronavirus HCoV-O43 utilize sialic acid moieties as receptor components. Therefore, the potent sialic acid biosynthesis inhibitory function of HCQ/CQ was marked as crucial for its broad antiviral spectrum activities [110]. Attenuated binding of SARS-CoV in CQ treated cells in vitro may substantially implicate the role of CQ in interrupting the glycosylation of host cell receptor, *viz.,* ACE2 in Vero E6 cells [15] (Figure 3A).

The second important aspect of the HCQ/CQ mode of action is inhibition of virus replication by abolishing the pH-dependent endosome-mediated viral entry at an early step (Figure 3A). Of note, a CQ-dependent increase in endosomal pH impacts cellular iron metabolism and restricts its release from transferrin in the endosome that leads to declined intracellular iron concentration [111]. This iron deficiency alters the function of several proteins/enzymes, primarily impacting the cellular replication and transcription machinery [111,112]. Early reports of HCQ/CQ increasing endosomal pH and stalling viral replication came from enveloped viruses, *viz.,* Chikungunya virus (CHIKV) or Dengue virus (DENV) [113,114]. Prophylactic treatment of CQ to Vero E6 cells in vitro before virus exposure was shown to alkylate endosomal pH and attenuate viral infection effectively [115]. The underlying mechanism of the above HCQ/CQ activity included inhibition of endocytosis, shift in endosomal pH, and impaired virus–endosome fusion [116]. The impact of HCQ/CQ was also seen on the binding of SARS-CoV to its DC-SIGN receptor [116]. The function of HCQ or CQ in the endosome is suggested to impact on the activation step where acidic pH facilitates viral and endosomal membrane fusion and subsequent SARS-CoV genome release into the cytoplasm [117] (Figure 3A). The function of the lysosomal compartment that retains acidic pH in viral membrane fusion and release of its genome is compromised due to the activity of HCQ/CQ weak bases [118]. The inhibitory effect of CQ on membrane fusion and uncoating was also shown to impair the replication of the hepatitis A virus [119].

The role of these quinolones is further implicated in impairing the post-translational modification of newly synthesized viral proteins. This process also requires low pH for optimal proteolytic enzymes and glycosyltransferases activities in the endoplasmic reticulum (ER) or at the adjoining trans-Golgi network vesicles (Figure 3A). The reports investigating anti-retroviral activities of CQ attributed its inhibitory effect to the gp120 envelope protein glycosylation, producing non-infectious newly synthesized human immunodeficiency viruses (HIV) proteins [120]. Similarly, CQ interferes in proteolytic processing of the flavivirus prM glycoprotein of the Dengue-2 (DENV-2) virus [121], thereby impairing its infectivity. The impact of CQ on the post-translational modification process was also attributed to impair budding of the herpes simplex virus (HSV) by accumulating the unprocessed HSV-1 virions in the trans-Golgi network [122]. Of note, animal (murine/feline) coronavirus M protein was earlier shown to determine the intracellular budding site for its virions that is reflected in its congregation in the Golgi complex [123]. Its assembly beyond the site of budding suggests a possible mode of CQ action from its antiviral activity in SARS-CoV-2 replication inhibition. A recent report finding a trans-Golgi network localization signal in the C-terminal domain of M protein in the MERS-CoV virus [124] essentially reaffirmed the regulatory role of M protein in intracellular budding of virions and therefore marked its potential as a drug for the potential effect of quinolones.

### 4.3. SARS-CoV-2, ACE2, and HCQ-Mediated Immunomodulatory Response

The immunomodulatory and anti-inflammatory activities of HCQ were recognized earlier due to its benefits in autoimmune diseases such as systemic lupus erythematosus (SLE) and rheumatoid arthritis (RA) [90]. At the molecular level, HCQ was shown to impair antigen processing in lysosome by antigen-presenting cells that reduce the recruitment of T-cells and expression of pro-inflammatory cytokines, *viz.*, IL-6 and TNFα [125] (Figure 3B). Multiple reports earlier underlined the anti-inflammatory activity of CQ/HCQ that include its inhibitory effect on IL-1β in THP-1 cells [126], IL-1, IL-6, and TNFα cytokine expression in monocytes/macrophages [125,127]. In the Dengue-2 virus-infected U937 cells, CQ was shown to inhibit IFN α, IFN β, IFN γ, IL-6, IL-12 and TNFα gene expression [128]. CQ/HCQ-mediated reduced secretion of pro-inflammatory TNFα cytokine was particularly validated in a murine macrophage cell line [129], mouse peritoneal macrophages [130], and also in human peripheral blood mononuclear [131] and whole blood cells [132]. TNFα, by activating monocytes, facilitates neutrophil extravasation by relieving tight junctions that further stimulate the expression of leukocyte adhesion molecules (LAM) aligned through human vascular endothelial cells [133]. Therefore, CQ/HCQ –led inhibition of TNFα is a significant event in its immunomodulatory activity. Besides inhibiting the TNFα production of activated monocyte-macrophages, CQ/HCQ was also found to reduce the expression of TNF receptors (TNFR 1 and 2) at the human monocytic cell surface, decreasing monocyte activation and leukocyte extravasation, thereby impairing TNFR-driven TNFα signaling [134] (Figure 3B).

Although immunomodulatory responses of HCQ are evident, its activities were not considered immunosuppressive and also showed no association with an elevated risk of infection [135]. The clinical outcome from multiple patient cohorts with rheumatologic disease showed a lack of immunosuppressive activities of HCQ, even in the long run, that could potentiate risk of any infection [135,136,137]. In the context of viral infection, HCQ was shown to impact on the innate immune response by disrupting vesicle acidification as an antiviral activity. A usual innate immune response of the host to SARS-CoV-2 comprises suppression of type I interferon. Toll-like receptor (TLR)-7 was recently shown to be involved in recognizing the SARS-CoV-2 RNA and subsequently stimulating the innate immune function in COVID-19 patients [138]. HCQ was shown to reduce the affinity of TLR-7 & TLR-9 to viral genome/RNA by raising endosomal pH leading to the restricted release of key cytokines, e.g., INFs, IL-6, & IL-12 (Figure 3B). Of note, no impact of CQ on MyD88-dependent signaling was observed, but its modulation by SARS-CoV was suggested to provide benefits in a murine challenge model [139]. HCQ also impairs cGAS (cyclic GMP-AMP synthase) function, which is essential for the production of type I interferon (IFNβ) and can be activated by RNA/DNA dependent mechanism [140] (Figure 3B). While SARS-CoV activates cGAS/STING, SARS-CoV-2 was found to be highly responsive to IFNβ [141,142]. Furthermore, HCQ is shown to attenuate the cytotoxic function of NK cells by controlling perforin processing to its active form [143]. This evidence suggested the involvement of HCQ/CQ in a modulation of the innate immune response in the host that is of significant clinical value. However, a precise readout of its molecular activity against SARS-Cov-2 warrants further careful investigation.

Multiple reports also revealed the effect of HCQ on the adaptive immune response. HCQ-induced increase in endosomal pH affects the processing and presentation of viral antigen that further attenuates T- and B- cell activation. CQ/HCQ treatment was also shown to decrease the count of prolific T-cells and control differentiation for Th1 and Th17 [144,145]. Interruption of antigen presentation by CQ/HCQ restricts activation of CD4 helper T-cells marked by CD154 expression, leading to reduced IL-6 and TNFα production [146]. CQ treatment causing inhibition of autophagy during T-cell activation was found to reduce the T helper cell’s response to antigen re-presentation, its proliferation, and IL-2 production [147]. Further, p38 mitogen-activated protein kinase (MAPK) inhibitory activity of CQ in the human monocytic cell line (THP-1) [126] affirmed results of an earlier finding that showed CQ-induced control on viral replication involves p38 MAPK inhibition [94] (Figure 3B). These results are critical in light of the fact that viruses require cell activation via MAPK signaling to achieve their replication cycle [148]. Although the available data so far primarily hints at the role of HCQ/CQ in attenuating the host’s innate immune and adaptive immune responses and reducing the collection of T-cells and B-cells produced in response to SARS-CoV-2, efforts to elucidate the molecular mechanism of CQ/HCQ’s activity in a case-specific manner, along with dosing, duration, and stage of disease, warrant careful further investigation.

## 5. ACE2, HCQ, and Clinical Outcomes: Assessing Cardiovascular Risk and Benefits

ACE2 has been central to SARS-CoV-2 pathology in the ongoing COVID-19 outbreak. Besides serving as a key component of RAS signaling in the cardiovascular system, several factors were shown to affect ACE2 functioning and, therefore, could impact on the clinical outcome of COVID-19 patients. Taking ACE2 into account, there are growing concerns about the ongoing repurposing of CQ/HCQ at enormous scale in clinics, insisting on the assessment of potential risk factors affecting ACE2 and HCQ repurposing regarding cardiovascular function. In this section, we discuss these aspects, readouts from available clinical outcomes, and the state of ongoing therapeutic regimes in current clinical trials.

### 5.1. ACE2 and Potential Cardiovascular Risk Factors

**Figure 4:**
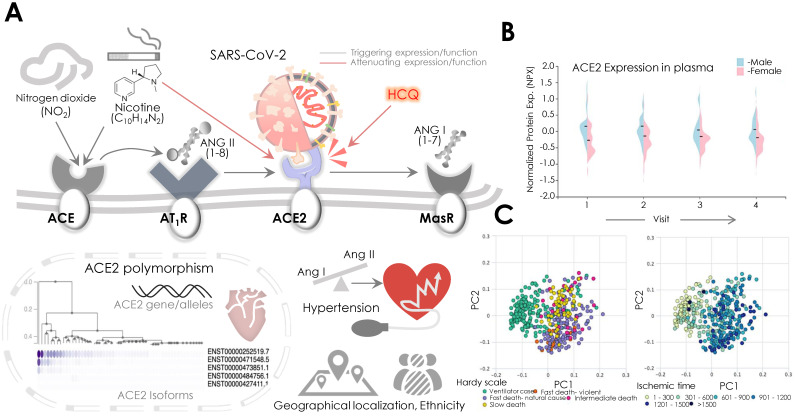
SARS-CoV-2, ACE2, and potential cardiovascular risk factors: assessing the vulnerability of COVID-19 infection. (**A**) Schematic diagram showing the risk of NO2 and nicotine in the modulation ACE2 expression, wherein levels of ACE2 and ratio of Ang II/Ang 1–7 determine the cardiovascular pathology. NO2 might increase, while nicotine might decrease the ACE2 levels, and this could alter the ratio of Ang II/Ang 1–7 in the heart triggering hypertension and risk of SARS-CoV-2 infection. Other potential factors that could potentially alter ACE2 expression include its genetic polymorphism, geographic localization, ethnicity, age, gender, and varied protein stability. (**B**) Graph (violin plot; image credit: Human Protein Atlas) shows the normalized protein expression of ACE2 levels in the blood plasma samples of the control males and females, where a relatively higher ACE2 expression can be seen in males than the females. (**C**) Graphs showing a lower ACE2 protein stability during death (on Hardy scale) and ischemia (calculated aa ischemic time) (image credit: Human Protein Atlas).

SARS-CoV-2 binding to ACE2 is suggested to cause loss of the latter and also alters its function, which eventually develops into the pathophysiology of cardio-respiratory failure [149]. Results from animal studies showed that loss of ACE2 promotes reactive oxygen species (ROS) production via NADPH oxidase 2 activation, while recombinant ACE2 administration was shown to attenuate Ang II function in TGFβ1 and collagen production [150]. Similarly, the expression of recombinant ACE2 was found to diminish the risk of pulmonary artery hypertension pathophysiology [151]. Of note, the inability of the loss of ACE2 function to manage the deleterious effects of Ang II was found to impair cardiac and pulmonary structure and function [152]. It is, therefore, argued that Ang II receptor inhibitors/blockers may potentially serve a cardio-protecting function in the later phases of COVID-19 disease. A recent study comparing circulatory levels of Ang II in healthy controls and COVID-19 patients demonstrated its significantly higher expression in the latter, which was found to be consistent with lower ACE2 levels [41]. These results thereby conferred the crucial role of ACE2 in balancing Ang I and Ang II levels. Of note, Ang II circulatory levels correlated well with viral load and negative cardio-respiratory function in the SARS-CoV-2 patient cohort [41]. The role of PARP in the modulation of ACE2 was also implicated in hypertensive rats, where inhibition of PARP could enrich ACE2 protein levels [153]. These data could explain the high prevalence (15–40%) of hypertension in COVID-19 patients [2,154] that increases further with disease severity [154]. Therefore, it conferred a crucial role to ACE2 and its function as a potential risk factor impacting CV function in COVID-19 patients (Figure 4).

Analyses of environmental and lifestyle-related factors were earlier shown to impact on ACE2 expression and function [155,156,157,158]. Results from animal studies showed a 100-fold increased ACE2 activity post NO2 exposure that also revealed a higher Ang II binding to its receptor [155,156]. Therefore, the role of ACE2 expression with Ang II binding to the AT1R was observed (Figure 4). These findings were confirmed by a population-based, cross-sectional survey that revealed an increased risk of hypertension with elevated exposure to NO2 in a population in China [159]. Data from the 2003 SARS epidemic further marked a positive association between air pollution and patient mortality in the Chinese population [160]. In the context of air pollution/NO2 levels, lockdown measures in the ongoing COVID-19 pandemic are suggested to benefit health, but high COVID-19 mortality was reported in areas with high NO2 pollution. Smoking was also suggested to be a key factor that may potentiate susceptibility to SARS-CoV-2 infection. Nicotine increases the expression of detrimental ACE, while reduced levels of compensatory ACE2/Ang (1–7) receptor axis were concurrently observed [157] (Figure 4). Although HCQ-mediated inhibition of ACE2 receptor glycosylation and the action of nicotine exerts control over ACE2′s SARS-CoV-2 binding, a recent clinical meta-analysis data denied association of latter with disease severity [157] (Figure 4).

The ACE2 gene is localized at the X chromosome and shows polymorphism (Figure 4). A significant correlation between ACE2 polymorphism and incidences of arterial hypertension is reported for women and associated with different ethnicity, race, and locality in Han Chinese men [161,162]. The ACE2 polymorphism distribution in the Chinese population varied with regions and was found to be associated with different blood pressure responses, while northern regions in China had an elevated response compared to the southern regions. This polymorphism was suggested to be a result of different climatic conditions, acquired by adaptive selection in populations over the generations [161,163]. The ACE1 and ACE2 polymorphism in Brazilian patients was also found to be associated with hypertension [164]. Furthermore, in Asian populations, ACE2 polymorphism was found to be correlated with the prevalence of cardiovascular comorbidities [71]. However, no evidence of its correlation with different susceptibility to SARS-CoV-2 infection or its severity is yet known. The geographical and ethnic distribution of ACE2 polymorphisms is also suggested to vary susceptibility to SARS-CoV-2 infection (Figure 4).

The factors that are apparently found to be linked with worse clinical outcomes in COVID-19 patients include the patient’s age and gender [2]. Earlier, results from the animal study suggested an age-dependent decline of ACE2 levels in the lungs. However, a recent observation-based prospective report analyzing ACE2 activity in bronchoalveolar lavage fluid lacked any significant correlation with age [165]. Besides examining ACE2 clinical outcome, Guan et al. clearly showed that older patients (mean age- 63 years; range 53–71) are more prone to experience intensive care (ICU admission, requiring ventilation), or fatality than younger patients (Mean age- 46 years, range 35–57) [2]. A relatively higher (50–80%) susceptibility of males to COVID-19 complications among hospitalized patients was also observed [5,6,7,8,166]. To check out the disparity of ACE2 level in males and females, we retrieved circulatory ACE2 expression (in the blood plasma, from healthy males and female controls) from the Human Protein Atlas (HPA; http://www.proteinatlas.org) (Figure 4). Normalized protein expression in plasma showed relatively higher levels of ACE2 in males than females (Figure 4), whereas analyses of protein levels during death and ischemia showed the distribution of ACE2 levels across Hardy scale and ischemic time (Figure 4).

### 5.2. HCQ Therapeutics and its Impact on Cardiovascular Function

The prophylactic and broad-spectrum benefits of HCQ/CQ in the absence of a SARS-CoV-2 specific antiviral or vaccine encouraged their large-scale clinical repurposing during the ongoing COVID-19 crisis [167]. Besides the growing therapeutic or ethical concerns about their yet unproven efficacy against SARS-CoV-2, potential risks of this medication should also be carefully assessed before clinical prescription [168,169]. The potentially detrimental effects of HCQ on cardiovascular function are known as primitive clinical outcomes. HCQ/CQ is known to produce mild cellular and cardiac toxicities. A systematic review assessing cardiac complications in HCQ/CQ treated patients for an extended period revealed conduction disorders as the leading side effect [170]. Other unfavorable cardiac outcomes included hypertrophy, heart failure, hypokinesia, valvular dysfunction, and pulmonary arterial hypertension. However, the above adverse outcomes improved in a significant number of patients (44.9%) upon HCQ/CQ withdrawal, while the remaining had irreversible events (12.9%) or mortality (30.8%) [170]. Cardiac conduction disorders are the leading cause of arrhythmia that underlined the proarrhythmic activity of HCQ/CQ, and it was suggested to inhibit the cardiac inward rectifier K+ current (Kir/IK1) and subsequently to induce lethal ventricular arrhythmia. These effects were partly seen with the attenuated human ether-à-go-go related gene (hERG) and Kir2.1 potassium channel activity that may be acquired at a low HCQ/CQ concentration [171,172]. The clinical readouts of HCQ/QC usage exhibit signs of QTc prolongation and risk of ventricular arrhythmias [173]. Although the occurrence of QTc prolongation in the setting of HCQ/CQ is yet to be interpreted correctly, its ECG readouts need to distinguish HQ outcome carefully to avoid a potential overlapping with existing cardiovascular comorbidities in COVID-19 patients. In a cohort of healthy participants, lower (600 mg) and higher (1500 mg) doses of CQ were shown to impact by causing an average 16 ms (95% CI: 9–23 ms) and 28 ms (95% CI: 18–38 ms) increase in QTc respectively [174], while the most significant QTc prolongation occurred four hours after being given the second dose. A combination of HCQ and azithromycin (AZM) for SARS-CoV-2 treatment was shown to significantly prolong the QTc interval over time in a cohort of 84 patients, where 18% showed a QTc increase by 40–60 ms, and 12% QTc >60 ms, while 11% overall showed QTc >500 ms, reflecting the risk of arrhythmia [175]. Amid contrary clinical outcomes of HCQ and AZM usage, where Gautret et al. showed the benefit [22], and Molina et al. [23] denied such effect of its treatment with COVID-19 patients, prescription of HCQ and AZM usage might impose further cardiovascular risk in the outpatient setting. In a retrospective study enrolling the population receiving HCQ for rheumatic disease, an increased risk of cardiovascular mortality in HCQ and AZM treated group over the HCQ and amoxicillin (AMX) treated group was observed [107]; however, overall mortalities were indifferent. On a similar note, application of lopinavir/ritonavir, a protease inhibitor that is frequently used for treating HIV infection and has exhibited an in vitro activity against SARS-CoV, showed no benefit/decrease in SARS-CoV-2 viral load in a 14 day open-label randomized trial [176], yet it is still being used with or without CHQ in some settings against COVID-19.

### 5.3. HCQ Repurposing and Heart: Therapeutic Regimes in Current Clinical Trials

Amid accelerated repurposing of HCQ/CQ for COVID-19, concerns of their safety in clinical practice are continually growing. An array of unsatisfactory clinical studies (patient cohort, perspectives, case reports) by date, showing mixed positive or negative results further made it crucial to determine the clinical outcome of CHQ/CQ repurposing. As a result, numerous randomized clinical trials evaluating the suitability of HCQ/CQ for COVID-19 were proposed and initialized worldwide. At present, out of 1717 registered clinical trials of COVID-19 at US NIH’s National Library of Medicine portal (https://www.clinicaltrials.gov/ct2/home) from all over the world, 206 studies are evaluating the prophylactic and therapeutic efficacy of HCQ/CQ. While surveying trials for an additional term “cardiovascular or heart”, 13 trials were found to be explicitly testing the efficacy of HCQ with an emphasis on cardiovascular safety in pre- and post-treatment settings. Table 3 lists all of these studies, and further information can be accessed at NIH’s ClinicalTrial.Gov portal.

Studies from China, including those of Wang et al. [19] and Gao et al. [24], earlier supported the repurposing of HCQ/CQ for COVID-19 treatment, by stating HCQ’s superiority over control treatments in shortening the disease course. Presently, out of 666 ongoing clinical trials in China on COVID-19, 13 trials are evaluating the use of HCQ for COVID-19, further information of which can be accessed at the ChiCTR portal (http://www.chictr.org.cn/enindex.aspx). Besides the reports backing CHQ repurposing [19,22,24], multiple hospitals/institutes worldwide have constituted guidelines for HCQ usage and assimilated instructions provided by the Centers for Disease Control and Prevention (CDC, USA) (available at https://www.cdc.gov/coronavirus/2019-ncov/hcp/therapeutic-options.html) describing the treatment regime and clinical relevance of HCQ in the COVID-19 pandemic. Recently, a clinical study from the University of Oxford and the National Health Service (NHS, U.K.) revealed “no beneficial effect” of HCQ in an inpatients randomized trial. Subsequently, this finding and existing data on HCQ led WHO to halt its Solidarity Trial that was planned to estimate its efficacy, along with other potential treatment arms. Furthermore, citing lack of a clear HCQ benefit, NIH also ceased its ORCHID trial after an interim review by an independent data monitoring committee, whereas a lack of active enrollment and safety concerns further paused clinical trials of generic HCQ makers Novartis and Sanofi, respectively. Although these recent developments dashed hopes of HCQ utility for COVID-19 patients, several institute or investigator-backed trials are still underway and may further shed light on HCQ’s function.

## 6. COVID-19, ACE2, and HCQ: Consideration and Recommendations

Preventive measures are the best approach against COVID-19. As noted, a higher level of ACE2 expression is associated with susceptibility to SARS-CoV infection *in vitro*, suggesting that the upregulated level of ACE2 promotes the risk of COVID-19 [177]. Ang II Receptor Blockers (ARBs) and ACE inhibitors (ACEIs) are promising candidates available for the treatment of CVD. Various studies suggest that ARBs and ACEIs increase ACE2 expression and inhibit ACE1 or block AT1R [178]. For COVID-19 patients, the termination of ARBs/ACEIs are not recommended as they block the RAS and protect patients against CV complications. Exogenous administration of recombinant ACE2 may be the most promising alternative to treat COVID-19 patients. Moreover, HCQ and AZM potentiated severe complications for patients with CVDs, which includes cardiac irregularities, e.g., arrhythmia, long QT syndrome, polymorphic ventricular tachycardia, along with increased risk of mortality. The combination of these two drugs on QT or arrhythmia has not yet been tested. The recommended dose for HCQ is 400 mg twice daily, followed by 200 mg twice daily for four days. Recommended dosage for azithromycin is 500 mg twice daily for five days, and clinical trials for these agents at various doses are in progress [179,180,181]. These doses are not recommended for children less than 12 years, and pregnant or lactating women. Initial results suggest HCQ benefits in reducing in-hospital duration, decreasing pneumonia severity, and in rapid virus clearance [24]. However, these doses should be administered under close medical supervision. ECG monitoring is recommended to observe cardiac arrhythmias, including QT prolongation, atrioventricular blockage, and Torsade de Pointe. The American Heart Association (AHA), American College of Cardiology (ACC), and the Heart Rhythm Society (HRS) jointly published guidelines for health care professionals, which include measures to reduce or mitigate CVD risk in COVID-19 patients. These measures included: (1) withdrawal of HCQ and azithromycin in patients with baseline QTc prolongation (QTc, ≥500 ms), (2) ECG/QT interval monitoring, (3) avoidance of other QTc prolonging agents whenever feasible, and (4) correction of hypokalemia >4 mEq/L and hypomagnesemia >2 mg/dL. Standard quality care is a must for patients while testing the efficacy of HCQ/CQ for COVID-19. While these agents may work as monotherapy or in combination, one needs to consider the effect of these medications on COVID-19 patients with existing CVD. Given the fact that present knowledge is inadequate and lacks a clear explanation of the efficacy of HCQ/CQ in preventing virus transmission, especially for healthcare professionals, we believe that results from clinical trials will provide more information that will be crucial in curbing the risks involved in the COVID-19 pandemic.

## 7. SARS-CoV-2, ACE2, and HCQ: The Way Forward

The ongoing COVID-19 pandemic has considerably stressed the global healthcare system and pushed it to a crisis, while now, with time, we are started learning to live with it. Most importantly, the last three months have tested the competence, coordination, and policymaking of healthcare sectors across the globe and exposed our preparedness in the hour of crisis.

HCQ/CQ repurposing, beyond anecdotal benefits, may buy us time to develop targeted therapeutics against COVID-19. A SARS-CoV-2 specific vaccine or antiviral is more likely to succeed and may fill the void, rather than anecdotal alternatives. The anecdotal HCQ repurposing should not be rendered as a cure. Therefore, it demands randomized controlled trials at earliest to elucidate its clear and safe benefits regarding COVID-19. Voluntary participation in such ongoing clinical trials of controls and persons with CV comorbidities worldwide may help us in grasping clinical outcomes early and may swiftly determine HCQ/CQ efficacy and its clinical regimes. Importantly, to work out a safer HCQ regime for elderly or patients having CV comorbidities, a clear understanding of the impact of ARBs and ACEIs is critically required and therefore this should also be tested in clinical trials. This may help us mitigate the impact of CV comorbidities and their risks in the course of an adopted therapeutic regime. Analysis of the patient’s RAS phenotype by its expression and function profiling, particularly for ACE2, before starting the treatment may also predict the critical involvement of ACE2 in SARS-CoV-2 severity and could tell us if ACEIs or ARBs could be of any help in the patient’s recovery. It may also further help us understand in the future the risk of SARS-CoV-2 infection in the population having preexisting CVDs and high ACE2 levels. In the light of available evidence, more protection is desirable for patients with CV comorbidities, and thus they could be preferentially immunized with a COVID-19 vaccine in the future. Therefore, this emphasizes the need to develop a SARS-CoV-2 -specific vaccine at the earliest.

Another critical aspect that needs to be carefully addressed in the future is a follow-up on COVID-19 survivor’s health. Elevated systemic inflammatory and incessant pro-coagulant activity persist in pneumonic patients even after discharge from the hospital. Clinical data suggest an association of pneumonia with increased risk of CDVs up to a decade after follow-up [182], which postulates a likely scenario for SARS-CoV-2 respiratory and CV infections. Similarly, a metabolic study showed disruption of lipid metabolism for twelve years after the clinical survival of 25 SARS patients [183]. Although factors including viral phenotype, severity, baseline characteristics and long-term prognosis may diversify the follow-up outcome, it is crucial to serially follow-up survivor’s health to predict the prognostic outcome in COVID-19 patients, especially those with preexisting CV comorbidities.

## Figures and Tables

**Table 1:** Pre-clinical readouts from the key in vitro studies investigating therapeutic efficacy of HCQ against SARS-CoV-2. HCQ, Hydroxychloroquine; EC50, Effective concentration; AZM, Azithromycin.

Investigation/References	Cell Systems	Drug, Concentration, and Assay Time (h)	Study Control	Key Findings/Comments
Yao et al. 2020	Vero E6 cell (Origin-African green Monkey)	CQ and HCQ 0.032, 0.16, 0.80, 4, 20, & 100 µM 2 h	-	-HCQ showed better SARS-CoV-2 inhibitory activity than CQ. -An extended incubation period may produce greater anti-viral effect
Liu et al. 2020	Vero E6 Cells	CQ and HCQ 0.068, 0.21, 0.62, 1.85, 5.56, 16.67, and 50 µM 1 h	PBS (Phosphate buffer saline)	-HCQ inhibited the steps including infection/entry and post-infection -At the higher viral replication rate, anti-viral efficacy of HCQ found to be lesser than of CQ
Wang et al. 2020	Vero E6 Cells	CQ and others * 0.01, 0.05, 0.1, 0.5, 1, 5, and 10 µM 1 h	DMSO	-HCQ inhibited the viral activity at low µM conc. (effective conc. EC50 = 1.13 μM) -CQ effectively inhibited SARS-CoV-2 infection in vitro
Andreani et al. 2020	Vero E6 cells	CQ- 1, 2 or 5 μM associated with 5 or 10 μM for azithromycin.	-	Combination of hydroxychloroquine and azithromycin has a synergistic effect in vitro on SARS-CoV-2 at concentrations
Keyaerts et al. 2004 (*Earliest report from the SARS-CoV)	Vero E6 cell	CQ 0, 0.8, 4, 20, & 100 µM 8 h to 3 days	-	-CQ potently inhibits SARS-CoV activity at a lesser (8.8 ± 1.2 μM) concentration than its cytostatic activity (261.3 ± 14.5 μM) -Addition of CQ even after 5 h of SARS-CoV infection could yet be inhibitory active

**Table 2:** Characteristics of HCQ therapeutic regimes and their outcomes in key comprehensive clinical studies.

Investigation/Reference	Investigation Type/Design	Patients (Total No)		Regimes	Severity of COVID-19 Disease	Results/Key Findings	Comment	Location	Limitation
		Con	HCQ						
Chen J et al. (2020)	Randomized and controlled trial	15	15	HCQ- 400 mg for 5 days	6–7 days symptomatic patients, unclear severity	Indifferent outcomes in groups. By day 7, no significant change in conversion rate (86.7% vs 93.3%) observed.	Patients were tested negative for COVID-19 at 2 weeks	Shanghai, China	Smaller sample size. Not peer-reviewed, availability in Chinese language
Gautret P et al. (2020a)	Open-label trail, Non-randomized, Non-blinded	16	26	HCQ- 600 mg for 10 days	Asymptomatic patients-17%, Patients with respiratory symptoms- 61%, Chest CT pneumonia +ve patients- 22%	Unadjusted results showed significantly reduced viral titer at day 6 (HCQ-70% vs. con 12.5%, PCR based, *p* < 0.01)	Exclusion of 6 patients from data (1- died, 1- withdrew, 3 needed ICU admission, 1- lost follow-up)	Marseille, France	Study design, Smaller sample-size, Exclusion of 6 patients, inconclusive long-term outcomes
Molina JM et al. (2020)	Prospective open-label investigation	0	10	HCQ- 600 mg for 5 days + AZM 500 mg × 1, then 250 mg	10 patients out of 11 were on supplemental oxygen	8 patients out of 10 were positive at day 5–6 (nasopharyngeal swab) (80%, 95% CI: 49–94)	Patient died-1, Patient transferred to ICU-2, Patient had no further HCQ post prolongation of QTc-1	Paris, France	Smaller sample size. Not peer-reviewed.
Chen Z et al. (2020)	Parallel-group trail Randomized	31	31	HCQ- 400 mg for 5 days	Mild illness was observed in CT confirmed pneumonia cases	- Clinical recovery and cough remission time reduced in HCQ group, while resolution of pneumonia was higher (80.60% vs. 54.8%) in the HCQ group.	Undefined status, 4 patients developed severe illness in the control group	Wuhan, China	Smaller sample size. Not peer-reviewed.
Gautret P et al. (2020b)	Open-label trail, Non-randomized, Non-blinded	0	80	HCQ- 600 mg for 10 days + 500 mg, followed by 250 mg AZM	Asymptomatic- 5%, Pneumonia cases- 54%, Patients with low national early warning score (NEWS) and mild disease- 92%	Decreased nasopharyngeal viral load at 7th (83% negative) and 8th (93%) days	Patients discharged from hospital - 65 (81.3%), Patients needed ICU admission- 1, Deceased- 1	Marseille, France	Design of the study, Smaller sample size. Not peer-reviewed. Short follow-up time period
Tang W et al. (2020)	Open-label, Multi-centric, Randomized, Controlled trial	75	75	HCQ- 200 mg for first 3 days, 800 mg for remaining days (total 2–3 weeks)	Patients with mild-moderate disease- 148. Patients with severe illness-2	HCQ showed no significantly higher negative conversion probability (85.4%) than control (81.3%) patients. Adverse effects were reported in HCQ group	Adverse events in control and HCQ group were reported in 7 and 21 patients respectively	Shanghai, Anhui, Hubei, China	Smaller sample size. Not peer-reviewed.
Million M et al. (2020)	Open-label trail, Non-randomized, Non-blinded	0	1061	HCQ- 200 mg (3 X/day) for 10 days + 500 mg AZM (day-1), followed by 250 mg for next 4 days	Patients had 20.5% and 2.2% moderate and severity scores respectively	In 10 day regime, good clinical results and virological cure were reported in 973 patients (91.7%). HCQ+AZM treatment before COVID-19 illness is safe and has low fatality rate in patients	Majority of patients had relatively mild symptoms at start (95%), therefore, only 10 patients (0.9%) transferred to the ICU, & 8 (0.75%) patients died	Marseille, France	Study design. Incomplete data on some patients. Unsynchronized diagnostic reports
Mahevas M et al. (2020)	Multi-centric, Non-Randomized, aim to emulate a target trial	97	84	HCQ- 600 mg for about ~7–8 days	Most patients had bilateral pneumonia, and 75% moderate or severe illness	No significant relief was observed in HCQ group as compared to control at day 7 in hospitalized patients. All comorbidities were less frequent in the HCQ group.	17 (20%) patients in the HCQ group, received concomitant AZM, while 64 (76%) received amoxicillin and clavulanic acid.	Créteil, Suresnes, Evry, and Paris, France	Not peer-reviewed. No randomization, Unbalanced prognostic variables across hospitals.
Magagnoli J et al. (2020)	Retrospective analysis, Non-randomized	158	97 (HCQ), 113 (HCQ+AZ)	-	All confirmed COVID-19 patients. No severity was specified	No evidence of HCQ either with or without AZM, lessen the risk of mechanical support in patients	Study comprises only men aged over 65 years, most black population	Virginia, and South Carolina, USA	Study design. Not peer-reviewed. Possibility of selection bias.
Mathies D et al. (2020) -Case report	Case report	0	1	HCQ- 400 mg for 1st day, then 200 mg for remaining 11 days	77-year-old COVID-19 positive patient with a heart transplant, moderate symptoms	Patient with existing dyspnea and dry cough, showed no further deterioration of the clinical state post HCQ medication. After 12 days, all negative	Patients survived and discharged from hospital after 12 days and had symptoms	Koblenz, Germany	-
Lane JCE et al. (2020) -Case series	A multinational, network cohort and self-controlled case series study	310, 350 (SSZ)	HCQ-956374 HCQ+AZM- 323122, HCQ+ AMX- 351956	- (variable)	16 patients had severe adverse events	No excess risk of severe events was identified when 30-day HCQ and SSZ (sulfasalazine) were compare. While, AZM + HCQ increased risk CVD and morality	cardiovascular complications in HCQ+AZM group are likely due to synergistic effects on QT length	Germany, Japan, USA Netherlands, Spain, & UK.	Not peer-reviewed. Potential risk of overlapping in patient datasets, variance in data

*Abbreviations:* HCQ, Hydroxychloroquine; CQ, Chloroquine; EC50, Effective Concentration; AZM, Azithromycin; SSZ, Sulfasalazine; AMX, Amoxicillin; CT, Computed tomography; NEWS, National early warning score; PCR, Polymerase chain reaction; ICU, Intensive care unit; QTc, Corrected Q and T wave. ** Last 2 rows in the dark enlist details of clinical case report/series.

**Table 3:** Table listing ongoing clinical studies investigating the efficacy of HCQ in therapeutic and prophylactic settings with an emphasis on cardiovascular concerns.

Trail Identifier	Study Title	Study Type/Design	Study Phase	Volunteers (Active)	Interventions/Drug(s)	Active Comparator	Primary Outcome	Location	Study Sponsor
NCT04371926	Prophylactic Benefit of HCQ in COVID-19 Cases with Mild to Moderate Symptoms and in Healthcare Workers with High Exposure Risk (PREVENT)	Interventional, Randomized	-	64	HCQ, 400 mg (day-1), then 200 mg for next 4 days (b.i.d.)	No-HCQ arm	Prophylactic Benefit of HCQ in patients and healthcare workers	-	Texas Cardiac Arrhythmia Research Foundation
NCT04341441	Will Hydroxychloroquine Impede or Prevent COVID-19 (WHIP COVID-19)	Interventional, Randomized	Phase 3	3000	HCQ, 400 mg (day-1), then 200 mg for a week (b.i.d.)	Placebo	Use of HCQ as a preventive therapy against COVID-19	United States	Henry Ford Health System
NCT04371744	AI for QT Interval Analysis of ECG From Smartwatches in Patient Receiving Treatment for Covid-19 (QT-Logs)	Observational, Cohort, Prospective	-	100	Not Applicable	-	Measurement of QTc using an AI and ECG data via smartwatches, compare to standard 12 leads ECG	Marseille, France	Assistance Publique Hopitaux De Marseille
NCT043329	Outcomes Related to COVID-19 treated with HCQ Among In-patients with Symptomatic Disease (ORCHID)	Interventional, Randomized	Phase 3	510	HCQ, 400 mg (day-1), then 200 mg for next 5 days (b.i.d.)	Placebo	Determine the COVID Ordinal Scale for patients on day 15	United States	Massachusetts General Hospital
NCT04353245	Study of Biomarkers in the Long-term Impact of Coronavirus Infection in the Cardiorespiratory System (PostCOVID19)	Observational [Registry], Case-Control	-	130	Arm treatment (HCQ + AZM)	-	Fibrosis on cardiac resonance and/or decreased functional capacity on ergo-spirometry	São Paulo, SP, Brazil	University of Sao Paulo General Hospital
NCT04372082	Hydroxychloroquine or Diltiazem-Niclosamide for the Treatment of COVID-19 (HYdILIC)	Interventional, Randomized	Phase 3	480	HCQ, 2200 mg (t.i.d.) during 10 days in addition to SOC; While niclosamide 500 mg × 4 at J1 then 500 mg (b.i.d.) + diltiazem 60 mg (t.i.d.) during 10 days	HCQ, Diltiazem & Niclosamide	Composite criteria- death, clinical worsening, and assisted-ventilation	Lille, France	University Hospital, Lille, France
NCT04361422	Isotretinoin in Treatment of COVID-19 (Randomized)	Interventional, Randomized	Phase 3	300	Isotretinoin, 13-cis retinoic acid 0.5 mg/kg/day b.i.d. for 1 month. Sham compa- HCQ 500 mg/12 h & other drugs	Active Comp: HCQ and other drugs+ isotretinoin	Viral clearance and COVID-19 virus load	Tanta city, Egypt	Tanta University, Egypt
NCT04374019	Novel Agents for Treatment of High-risk COVID-19 Positive Patients	Interventional (Clinical Trial), Randomized	Phase 2	240	HCQ 200 mg (t.i.b.) for 14 days. HCQ combination with AZM, Ivermectin, and Camostat Mesilate are also enrolled	-	Proportion of patients experiencing clinical deterioration	Kentucky, United States	Susanne Arnold, University of Kentucky
NCT04382625	Hydroxychloroquine in SARS-CoV-2 (COVID-19) Pneumonia Trial	Interventional, Randomized (Open Label)	Phase 4	120	HCQ 400 mg × 2 (800 mg) then 200 mg, t.i.b. (600 mg/24 h period) starting 8 h after 1st dose, total 14 doses over 5 days	-	Data collection, Change from Baseline Oxygenation on Day 1-5	Washington SU, USA	Kootenai Health, United States
NCT04333355	Safety in Convalescent Plasma Transfusion to COVID-19	Interventional, Open label	Phase 1	20	Convalescent Plasma	-	Adverse effects of administration of convalescent plasma	Mexico	Hospital San Jose Tec de Monterrey, Mexico
NCT04358068	Evaluating the Efficacy of Hydroxychloroquine and Azithromycin to Prevent Hospitalization or Death in Persons With COVID-19	Interventional, Randomized	Phase 2	2000	HCQ (200 × 2 mg day-1, then 200 mg × 2 for 6 days) + AZM (250 mg × 2 mg- Day 0, then 250 mg once daily for 4 doses (4 days)	Placebo	Proportion of patients’ mortality with COVID-19	San Diego, United States	National Institute of Allergy and Infectious Diseases (NIAID), USA
NCT04373044	Antiviral Therapy and Baricitinib for the Treatment of Patients with Moderate or Severe COVID-19	Interventional (Clinical Trial), Open label	Phase 2	59	1) HCQ, PO t.i.d., 2) lopinavir/ritonavir PO b.i.d., or 3) remdesivir.	-	Proportion of patients requiring invasive mechanical ventilation or dying	United States	University of Southern California, United States
NCT04349410	The Fleming [FMTVDM] Directed CoVid-19 Treatment Protocol (FMTVDM)	Interventional, Randomized	Phase 2, Phase 3	500	HCQ, 200 mg po q 8 hrs (600 mg qD) for 10-days, & HCQ regime with other drugs	-	Improvement in FMTVDM Analyzed by nuclear imaging	United States	The Camelot Foundation, USA

*Abbreviations:* HCQ, Hydroxychloroquine; CQ, Chloroquine; AZM, Azithromycin; SCO, Standard of care; b.i.d., bis in die (twice a day, for HCQ dose); t.i.d., ter in die (trice per day); QTc, Corrected Q and T wave; AI, Artificial intelligence, PO, Per os (Orally); ECG, electrocardiogram; FMTVDM, Fleming Method for Tissue and Vascular Differentiation and Metabolism.
